# The Role of Nutraceuticals in the Optimization of Lipid-Lowering Therapy in High-Risk Patients with Dyslipidaemia

**DOI:** 10.1007/s11883-020-00887-z

**Published:** 2020-09-18

**Authors:** Peter E. Penson, Maciej Banach

**Affiliations:** 1 School of Pharmacy and Biomolecular Sciences, Liverpool John Moores University, Liverpool, UK; 2 Liverpool Centre For Cardiovascular Science, Liverpool, UK; 3 University of Liverpool, Liverpool, UK; 4 Department of Hypertension, Medical University of Lodz, Rzgowska 281/289, 93-338 Lodz, Poland; 5 Polish Mother’s Memorial Hospital Research Institute (PMMHRI), Lodz, Poland; 6 Cardiovascular Research Centre, University of Zielona Gora, Zielona Gora, Poland

**Keywords:** Efficacy, Lipid disorders, Nutraceuticals, Safety, Therapy

## Abstract

**Purpose of Review:**

We aimed to summarize recent guidelines, position papers, and high-quality clinical research relating the use of nutraceuticals in the management of individuals at high risk of atherosclerotic cardiovascular disease.

**Recent Findings:**

It is essential that individuals at high risk of cardiovascular disease receive guideline-directed evidence-based therapies to reduce their risk of morbidity and mortality from cardiovascular events. Compared with conventional therapeutics, nutraceuticals have undergone relatively little investigation in randomized controlled trials. Thus, recommendations for nutraceuticals in international guidelines are rare, and nutraceuticals should not be used preferentially in place of statins. Nevertheless, recent position papers from the International Lipid Expert Panel and clinical evidence from studies of triglyceride reduction by polyunsaturated fatty acid administration demonstrate that nutraceuticals do have an important role in optimizing therapy in individuals at high risk of cardiovascular disease. Roles for nutraceuticals include as follows: (1) managing residual risk associated with lipids other than low-density lipoprotein cholesterol (LDL-C); (2) managing non-lipid-mediated residual risk; (3) optimizing LDL-C treatment in statin intolerance; (4) optimizing LCL-C treatment when add-on therapies for statins are not available; (5) as adjuncts to lifestyle for individuals at high lifetime risk of atherosclerotic cardiovascular disease (ASCVD). The strength of evidence for each of these applications is variable.

**Summary:**

In addition to guideline-directed therapeutics, nutraceuticals may have roles in optimizing preventative therapy and targeting residual risk in individuals at high risk of ASCVD. Application of Good Manufacturing Practice and randomized controlled trials when producing and evaluating nutraceuticals will expand the armoury of evidence-based agents for the prevention of ASCVD.

## Introduction

The term “nutraceutical” is a portmanteau derived from “NUTRient” and “pharmACEUTICAL”. The first recorded use of the term was in 1989 by DeFelice who defined a nutraceutical as “food, or parts of a food, that provide medical or health benefits, including the prevention and treatment of disease” [1, 2]. More recently, nutraceuticals have been distinguished from functional foods on the basis that the latter are “similar in appearance to conventional foods … consumed as part of a usual diet” [3] whereas nutraceuticals are “produced from foods but sold in pills, powders, (potions) and other medicinal forms not generally associated with food” [3]. Nutraceuticals, therefore, provide an opportunity to take advantage of biologically active, health-promoting molecules from nature, while retaining the control of dose, quality, and composition of formulations, in a similar manner to conventional pharmaceuticals. Unsurprisingly, then, nutraceuticals have elicited great enthusiasm from both patients and physicians for the prevention and treatment of a large number of diseases, including dyslipidaemias, the subject of this manuscript.

Atherosclerotic cardiovascular disease (ASCVD) is a major cause of morbidity and mortality worldwide. The World Health Organization Global Burden of Disease report estimates that 17.9 million deaths were attributable to cardiovascular disease in 2017 [4]. Despite this alarming statistic, considerable success and progress have been made in the identification and treatment of the risk factors for cardiovascular disease. The Framingham Heart Study has provided epidemiological insights into the causes of cardiovascular disease since its inception in 1948. Data from this study confirmed early observations by Gofman [5] that elevated low-density lipoprotein cholesterol (LDL-C) was associated with atherosclerotic cardiovascular disease and led to the development of the first risk prediction tools [6].

LDL-C (and other cholesterol-carrying lipoproteins which contain apolipoprotein B (apoB)) are prone to deposition in the walls of blood vessels. This process results in the development of atherosclerotic plaques which impede blood flow (resulting in angina and peripheral vascular disease). Plaque rupture exposes circulating platelets to tissue factors and results in intravascular coagulation, causing myocardial and cerebral ischaemia [7].

The causal role for LDL-C in atherosclerotic disease has been unambiguously demonstrated in evidence from hundreds of observational, interventional, and genetic studies [8–10]. Crucially, the risk conferred by LDL-C can be modified by drugs which reduce circulating LDL-C and cause upregulation of hepatic LDL receptors. Statins (inhibitors of 3-hydroxy-3-methylglutaryl-coenzyme A (HMG-CoA) reductase), a rate-limiting step in the mevalonate pathway of cholesterol production), have been available since the late 1980s. Randomized controlled trials (RCTs) have demonstrated that statins reduce the risk of cardiovascular disease by about one-quarter for each mmol/L (38 mg/dL) reduction of LDL-C during each year of therapy, with a lag time of only 1 year [11]. This relative reduction of risk means that the absolute expected benefit of LDL-C reduction depends upon the starting level of risk. Furthermore, individuals at high risk of atherosclerotic disease (who are the subject of this review) have the greatest expected benefit.

When optimal lipid-lowering cannot be achieved with statins in high-risk individuals, further lipid-lowering can be achieved with inhibitors of proprotein convertase subtilisin/kexin type 9 (PCSK9), an enzyme which promotes inactivation of LDL receptors. PCSK9 inhibition results in a reduction in atherosclerotic cardiovascular events, although the treatments are complex monoclonal antibodies (alirocumab and evolocumab) [12] or small interfering ribonucleic acid (siRNA, inclisiran) [13], are expensive, and are not widely available in all health systems.

Ezetimibe, an inhibitor of the Niemann-Pick C1-like 1 protein on epithelial cells, reduces cardiovascular events in combination with statin therapy [14], with the greatest absolute benefit being observed in individuals aged > 75 years [15]. Finally, bempedoic acid has recently been licenced by the Food and Drug Administration (FDA) in the USA. Bempedoic acid is an inhibitor of adenosine triphosphate-citrate lyase, an enzyme upstream of HMG-CoA reductase in the mevalonate pathway. Bempedoic acid appears to be safe and effective at lowering LDL-C, and outcomes trials are ongoing [16].

In light of this impressive array of lipid-lowering drugs, it might be suggested that there is little place for nutraceuticals in the management of patients at high risk of ASCVD. In fact, there are five clear roles which nutraceuticals can play:

1. Managing residual risk associated with lipids other than LDL-C
2. Managing non-lipid-mediated residual risk
3. Optimizing LDL-C treatment in statin intolerance
4. Optimizing LCL-C treatment when add-on therapies for statins are not available
5. Adjuncts to lifestyle for individuals at high lifetime risk of ASCVD

See Table 1 for an overview of nutraceutical use in these scenarios.

**Table 1:** A summary of potential uses for nutraceuticals according to the clinical scenarios outlined in this paper

	Clinical scenario	Nutraceuticals	Potential benefit	Evidence	References
1	Managing residual risk associated with lipids other than LDL-C	Icosapent ethyl (EPA)	↓Triglycerides and ↓CVD events	Large RCT	[17, 18]
		l-Carnitine	↓Lp(a)	Small RCTs	[19, 20]
		Coenzyme Q10	↓Lp(a)	Small RCTs	[19, 21]
2	Managing non-lipid-mediated residual risk	Plant sterols and stanols, red yeast rice, bergamot, berberine, polyunsaturated omega-3 fatty acids, and l-carnitine	↓Inflammatory markers	Animal studies and small RCTs	[22–24]
3	Optimizing LDL-C treatment in statin intolerance	Vitamin D	May ↓ severity of SAMS	Small RCTs	[25]
		Coenzyme Q10	May ↓ severity of SAMS	Small RCTs	[26]
4	Optimizing LCL-C treatment when add-on therapies for statins are not available	Armolipid Plus^®^	Nutraceutical polypill approach to ↓ LDL-C	Small RCTs	[27]
5	Adjuncts to lifestyle for individuals at high lifetime risk of ASCVD	All nutraceuticals listed above	↓ Risk factors in patients with a low 10-year risk, but appreciable lifetime risk of CVD	NA	[28]

*ASCVD* atherosclerotic cardiovascular disease, *CVD* cardiovascular disease, *EPA* eicosapentaenoic acid, *LDL-C* low-density lipoprotein cholesterol, *RCT* randomized controlled trial, *SAMS* statin-associated muscle symptoms

Furthermore, even when these conditions are not applicable, many patients will choose to take nutraceutical preparations because they are widely available (regardless of evidence or circumstances). Many individuals have strong beliefs that “natural” remedies are somehow better or safer than “conventional medicine”. Therefore, it is important that all those involved in treating any disease or condition have knowledge of available nutraceuticals in their field, and an appreciation of how these might interact with physiological processes, and other medicines the patient may be taking.

Large, outcomes-driven RCTs of nutraceuticals are rare. Because nutraceuticals are derived from natural products (which are not patentable in most jurisdictions) and because they do not usually emerge through the same developmental pathways as conventional pharmaceuticals, much of the evidence we have comes from mechanistic laboratory studies, and small studies in humans, usually employing biomarkers as their endpoints. An excellent, comprehensive, and detailed overview of the evidence for a wide range of nutraceuticals has been evaluated and published as a position paper by the International Lipid Expert Panel [22, 23]. It is essential that, wherever possible, individuals at high risk of ASCVD receive guideline-directed therapy based upon treatments which have demonstrated efficacy against hard cardiovascular outcomes in multiple, large RCTs. Nutraceuticals should not be recommended as alternatives to these life-prolonging therapies. Nevertheless, in several cases, there is good reason to believe that nutraceuticals may be able to optimize therapy. The remainder of this review will indicate how recent evidence demonstrates that lipid-lowering therapy in high-risk individuals can be optimized according to the five headings listed above. The focus will be predominantly on human studies using validated biomarkers and hard outcomes; however, we will indicate important early-stage research evaluating further roles for nutraceuticals in these settings. Much of this optimization aims to target “residual risk”: the risk that remains despite optimal management of LDL-C. In the recent CANTOS trial of canakinumab (discussed in more detail later), statins were taken by > 90% of participants, and median LDL-C was approximately 82 mg/dL (2.1 mmol/L) at baseline. Nevertheless, after 3.7 years of follow-up, 4.5 ischemic events/100 person-years occurred in the placebo group, indicating residual risk. Such residual risk is an important target for therapy, particularly in individuals at high risk of ASCVD. Some aspects of residual risk may be amenable to treatment with nutraceuticals.

## Nutraceuticals to Manage Residual Risk Associated with Lipids Other than LDL-C

The clear and definitive relationship between LDL-C and ASCVD and the success of statin therapy may have directed due attention away from other important circulating lipids for risk prediction and as treatment targets. Recent findings concerning nutraceutical-mediated triglyceride-lowering have renewed the focus on lipids other than LDL-C. Evidence from RCTs, observational studies, and meta-analyses strongly suggest that consumption of oily fish and long-chain omega-3 polyunsaturated fatty acids (PUFAs) improves risk factors and biomarkers associated with cardiovascular disease [19, 29, 30], in a manner consistent with the understanding of the biological actions of PUFAs, in particular the triglyceride-lowering effects of eicosapentaenoic acid (EPA) and docosahexaenoic acid (DHA) [31]. Because these active components can be chemically identified and standardized and appear to be closely associated with improvements in risk factors, PUFAs are an attractive target for nutraceutical development, and unusually for the field of nutraceutical research, three large, rigorous clinical trials have been published. The conflicting results of the three trials teach us important lessons about the management of cardiovascular disease in high-risk individuals and about the importance of employing the same rigour as used in the development and evaluation of nutraceuticals as we expect in the case of conventional pharmaceuticals.

In most countries, pharmaceutical preparations of “fish oil” or “PUFAs” are available for sale over-the-counter or on prescription. These preparations typically contain a variety of ingredients, often including EPA and DHA. The ASCEND trial was a placebo-controlled randomized two-by-two factorial evaluation of a once-daily dose one such preparation (460 mg EPA, 380 mg DHA—the composition recommended by the Nutrition Committee of the American Heart Association (AHA) [32]) and aspirin 100 mg/day in 15,480 participants with diabetes—an inherently “high-risk” population. The primary endpoint was a first serious vascular event (defined as a composite myocardial infarction, stroke, transient ischemic attack, or vascular death). After an impressive mean follow-up of 7.4 years, there was no significant difference between the treatment and placebo groups with respect to the primary outcome [33]. The VITAL study also investigated the effectiveness of a 460-mg EPA, 380-mg DHA preparation (in a two-by-two factorial placebo-controlled RCT with vitamin D_3_, 2000 IU per day). The trial was conducted in a primary prevention population and used a preparation containing the AHA recommended composition [32] (460 mg of EPA and 380 mg of DHA). The participants were 25,871 individuals, including men over the age of 50 years and women 55 years or older. The primary endpoints were a composite of major cardiovascular events (myocardial infarction, stroke, or cardiovascular death) and invasive cancer. Over a median of 5.3 years of follow-up, no significant differences were found between the placebo and treatment groups with respect to the cardiovascular composite endpoint [34].

Following these two “negative” trials, the REDUCE-IT randomized, placebo-controlled trial surprised some commentators when it reported that over a 5-year follow-up in 8179 participants, treatment with PUFA led to a 25% relative reduction (HR 0.75 95% [CI] 0.68–0.83 *p* < 0.001) in first events of a composite cardiovascular outcome (including cardiovascular death, myocardial infarction, stroke, coronary revascularization, and unstable angina). A subsequent analysis of REDUCE-IT data additionally showed that icosapent ethyl also reduced subsequent and total ischemic events [17]. The intervention was, however, very different from that employed in ASCEND and VITAL, consisting of a substantially larger dose (4 g) of highly purified EPA (icosapent ethyl). The study is of particular interest to the consideration of a “high-risk” population, as it included statin-treated individuals with cardiovascular disease or with diabetes and other risk factors, who had elevated triglycerides. Icosapent ethyl was not free of adverse effects. Treatment was associated with an increased risk of atrial fibrillation or flutter (3.1% vs. 2.1%, *p* = 0.004) and serious bleeding events (2.7% vs. 2.1%, *p* = 0.06) [18]. Nevertheless, this small increase in adverse effects is unlikely to outweigh the benefit of icosapent ethyl in reducing the risk of ASCVD in patients with elevated triglycerides. Interestingly, the STRENGTH trial, an outcomes trial of combined EPA and DHA in statin-treated patients with high levels of triglycerides and low levels of high-density lipoprotein cholesterol [35], was recently halted owing to futility [36]. Clearly the dose and composition of PUFA preparations is important in determining outcomes. Additionally, in a recent study which combined a systematic review and meta-analysis of RCTs and a Mendelian randomization, Mazidi et al. evaluated the link between omega-3 supplementation and CVD outcomes [37]. They showed a significant reduction in the risk of coronary heart disease (CHD) death (risk ratio [RR] 0.91, 95%CI 0.85–0.97, *p* = 0.010), major vascular event (RR 0.95, 95%CI 0.93–0.98, *p* = 0.001), non-fatal myocardial infarction (MI) (RR 0.89, 95%CI 0.83–0.95, *p* = 0.001), and all-cause mortality (RR 0.95, 95%CI 0.92–0.99, *p* = 0.025) with omega-3 interventions. Based on the data from Mendelian randomization, they observed that a genetically determined higher level of serum alpha-linolenic acid (ALA) had a negative impact on the CHD risk (IVW = beta: − 4.424, *p* = 1.1 × e−12) and MI (IVW = beta: − 2.081, *p* = 0.009). Interestingly, such significant changes were not observed for EPA levels [37].

Lipoprotein(a) (Lp(a)) is another relatively neglected lipoprotein which is both thrombogenic and atherogenic and which confers considerable risk of ASCVD when elevated [38, 39]. Circulating concentrations of Lp(a) are largely genetically determined; however, dietary and nutraceutical approaches to Lp(a)-lowering including l-carnitine and coenzyme Q10 have been evaluated and shown promise in clinical and experimental studies [40, 41]. If nutraceutical approaches to Lp(a) are shown to be effective in RCTs, the resulting products could serve an important role in optimizing lipid-lowering therapy in individuals at high risk of ASCVD. Evidence from RCTs suggests that in some individuals, statin therapy results in a modest elevation of Lp(a) [42, 43]. This small effect is unlikely to be of major concern, and any risk increase conferred by Lp(a) is likely to be outweighed many times over by the benefits of LDL-C-lowering. Nevertheless, if it were possible to reverse the Lp(a) elevation using nutraceuticals, this would help to optimize therapy.

## Managing Non-lipid-Mediated Residual Risk

Many therapeutic strategies can be employed to target the two most important risk factors for ASCVD, which are LDL-C [8–10] and blood pressure [44] (the latter is beyond the scope of this review). Other risk mediators have received less attention. Currently, the role of inflammation in the pathogenesis of ASCVD is a topic of intense research and public interest. Virchow [45] first posited in 1858 that inflammation was an important mediator in the pathogenesis of atherosclerotic diseases, and interest in the link between inflammation and ASCVD has continued to gather attention ever since [46, 47]. However, only recently have anti-inflammatory interventions been shown to reduce the risk of ASCVD in well-designed RCTs. The CANTOS trial demonstrated that treatment with canakinumab, a monoclonal antibody against the pro-inflammatory cytokine interleukin-1-β, significantly reduced cardiovascular events, without affecting plasma lipid levels [48]. However, the CIRT trial of methotrexate (methotrexate), which was intended to target the same inflammatory pathway (albeit at a different location), did not result in a reduction in cardiovascular events [49]. Finally, the COLCOT trial demonstrated and showed that colchicine, a potent anti-inflammatory agent with multiple modes of action, was effective at preventing major adverse cardiac events in the secondary prevention of ASCVD [50]. The failure of methotrexate to reduce cardiovascular events suggests that not all anti-inflammatory agents are equal with respect to ASCVD prevention, and further work is required to understand the particular anti-inflammatory pathways and molecules which need to be targeted to achieve benefit. Reduction of inflammatory markers has been demonstrated after treatment with a range of nutraceuticals including plant sterols and stanols, red yeast rice, bergamot, berberine, polyunsaturated omega-3 fatty acids, and l-carnitine. However, a limitation of many of these studies is that anti-inflammatory activity has been quantified by changes in C-reactive protein (CRP), a non-pathway-specific marker of inflammation, which provides insufficient detail about specific pathways being targeted [22, 23]. Therefore, it is hard to predict which agents might have a useful effect in atherosclerosis. Nevertheless, it is possible that as our understanding of the specific pathways involved in atherosclerotic inflammation is fully elucidated, nutraceuticals may become available to target these pathways.

## Optimizing LDL-C Treatment in Statin Intolerance

Statin intolerance is the condition whereby an individual has such severe side effects to statin side effects that that they cannot tolerate any dose of any statin (complete intolerance) or cannot tolerate guideline-directed dose of statins (partial intolerance). The vast majority (approximately 95% of individuals with statin intolerance) have partial intolerance and can tolerate lower doses of statins. A rigorous process of drug dechallenge and rechallenge, and consideration of the temporal relationship between drug administration and adverse effects must be carried out to determine whether the drug is really the cause of the symptom [51–53]. This is important because statin under-use and discontinuation is associated with poor clinical outcomes [54]. Furthermore, as much as 80% of reported adverse effects of statins may occur as a result of the Drucebo (DRUg + plaCEBO/noCEBO) effect, whereby the patient attributes a common symptom to a drug, because they expected the drug to cause that effect [55•]. In the case of statin intolerance, a position paper from the International Lipid Expert Panel recommends that nutraceuticals may be considered add-on therapies for statins [56] (see the “Optimizing LCL-C Treatment When Add-on Therapies for Statins Are Not Available” section). The International Lipid Expert Panel has also produced a helpful position paper relating to the use of nutraceuticals in statin intolerance [24••]. Of particular interest are products derived from red yeast rice, which contain naturally occurring lovastatin, although it can be tolerated by some statin-intolerant individuals [24, 57, 58]. Furthermore, supplementation with vitamin D [25] and CoQ10 [26] has been reported to reduce the severity of statin-associated muscle symptoms, and despite equivocal evidence for benefit, might be considered an approach to improve tolerance of statin therapy.

## Optimizing LCL-C Treatment When Add-on Therapies for Statins Are Not Available

Where statin therapy alone is insufficient to achieve optimal circulating concentrations, additional lipid-lowering therapies, including ezetimibe and PCSK9 inhibitors, are indicated. However, these may not be available in all cases (especially as the cost of PCSK9 inhibitors is substantially higher than for statins). If this is the case, then high-quality lipid-lowering nutraceutical preparations might be considered. One such interesting example is Armolipid Plus^®^ [27] which contains three naturally occurring molecules with evidence for lipid-lowering properties: red yeast rice, policosanol, and berberine. These are combined into tablets with folic acid, astaxanthin, and coenzyme Q10. The evidence supporting each of these ingredients individually and in combination has been comprehensively summarized in the International Lipid Expert Panel position paper on lipid-lowering nutraceuticals [22, 23]. In combining multiple active molecules into a single nutraceutical formulation, Armolipid Plus can be considered to be a nutraceutical polypill. In general, the advantages of polypills are that they aid compliance by simplifying dosing regimens, and by using low doses of several complimentary agents (rather than a larger dose of a single agent), adverse effects can be minimized [59].

## Adjuncts to Lifestyle for Individuals at High Lifetime Risk of ASCVD

When considering individuals at high risk, it is essential to consider the fact that most risk prediction tools use demographic data and physiological or biochemical measurements to predict the risk of cardiovascular disease over a defined period of time [28]. A variety of risk scoring systems have been developed. Categorization into graduated levels of risk is arbitrary but has important implications for the management of disease when guidelines direct therapies based upon these categories.

The European Society of Cardiology (ESC)/European Atherosclerosis Society (EAS) [60] guidelines use the SCORE 10-year risk calculation [60, 61]. In the UK, eligibility for lipid-lowering therapy is determined in part by the QRISK [62, 63]. The American College of Cardiology (ACC)/AHA guidelines base recommendations for statin therapy using the pooled-cohort equations to evaluate 10-year risk [64, 65]. Basing treatment decisions on 10-year risk might well be sensible for older individuals who are likely to carry several competing risks for morbidity and mortality. However, the approach may work less well in younger individuals. It is important to appreciate that age is a key contributor to the risk scores, and therefore, 10-year risk scores are likely to underestimate the lifetime risk of ASCVD in younger patients [66, 67]. Clinical guidelines which employ 10-year scores to initial therapy might overlook the benefits which could be accrued by treating a single modifiable risk factor (such as LDL-C), which, if untreated, is likely to confer harm at some point during the individual’s life [68]. Unfortunately, very little trial evidence is available to inform the optimal approach to these patients. Clearly, promotion of a healthy lifestyle and balanced diet is imperative, but may be insufficient to control elevated cholesterol deriving from endogenous production. Because of the low short-term risk, the paucity of evidence, and the possibility of adverse effects, statins may not be favoured by patients or physicians in this context (although they are likely to be beneficial).

## Conclusion

Optimization of ASCVD prevention is important in all patients, especially at those at high risk of cardiovascular disease. This must, wherever possible, involve guideline-directed use of evidence-based therapeutics at recommended doses. Nevertheless, such an approach reduces, but does not remove the risk of ASCVD. Nutraceuticals may have roles in optimizing preventative therapy, and targeting residual risk. The recent experience of triglyceride-lowering PUFAs, which had no effect on ASCVD in two trials, before showing remarkable risk reduction in REDUCE-IT is instructive. Clearly, the dose and composition of nutraceuticals must be as carefully controlled as is the case for conventional pharmaceuticals—and large, well-conducted, outcomes-driven RCTs must be used to evaluate their effectiveness. Furthermore, treatment effectiveness is likely to depend upon matching patient characteristics to the mechanisms of action of drugs—e.g., triglyceride-reducing icosapent ethyl has been shown to be effective in individuals with elevated triglycerides. Studies investigating the anti-inflammatory and LDL-lowering effects of nutraceuticals are generally less advanced. Nevertheless, we hope that rigorous application of Good Manufacturing Practice and appropriate evaluation of efficacy will expand the armoury of evidence-based nutraceuticals available for the fight against ASCVD.

## Abbreviations

ACC: American College of Cardiology
AHA: American Heart Association
ASCVD: Atherosclerotic cardiovascular disease
DHA: Docosahexaenoic acid
EPA: Eicosapentaenoic acid
EAS: European Atherosclerosis Society
ESC: European Society of Cardiology
FDA: Food and Drug Administration
LDL-C: Low-density lipoprotein cholesterol
NICE: National Institute for Health and Care Excellence
PUFA: Polyunsaturated fatty acids
RCT: Randomized controlled trial
